# Experimental Evidence for the Involvement of PDLIM5 in Mood Disorders in Hetero Knockout Mice

**DOI:** 10.1371/journal.pone.0059320

**Published:** 2013-04-08

**Authors:** Yasue Horiuchi, Maya Ishikawa, Nobuko Kaito, Yoshimi Iijima, Yoshiko Tanabe, Hiroki Ishiguro, Tadao Arinami

**Affiliations:** Department of Medical Genetics, Majors of Medical Sciences, Graduate School of Comprehensive Human Sciences, University of Tsukuba, Tsukuba, Ibaraki, Japan; Chiba University Center for Forensic Mental Health, Japan

## Abstract

**Background:**

Reports indicate that PDLIM5 is involved in mood disorders. The *PDLIM5* (PDZ and LIM domain 5) gene has been genetically associated with mood disorders; it’s expression is upregulated in the postmortem brains of patients with bipolar disorder and downregulated in the peripheral lymphocytes of patients with major depression. Acute and chronic methamphetamine (METH) administration may model mania and the evolution of mania into psychotic mania or schizophrenia-like behavioral changes, respectively.

**Methods:**

To address whether the downregulation of PDLIM5 protects against manic symptoms and cause susceptibility to depressive symptoms, we evaluated the effects of reduced *Pdlim5* levels on acute and chronic METH-induced locomotor hyperactivity, prepulse inhibition, and forced swimming by using *Pdlim5* hetero knockout (KO) mice.

**Results:**

The homozygous KO of *Pdlim5* is embryonic lethal. The effects of METH administration on locomotor hyperactivity and the impairment of prepulse inhibition were lower in *Pdlim5* hetero KO mice than in wild-type mice. The transient inhibition of PDLIM5 (achieved by blocking the translocation of protein kinase C epsilon before the METH challenge) had a similar effect on behavior. *Pdlim5* hetero KO mice showed increased immobility time in the forced swimming test, which was diminished after the chronic administration of imipramine. Chronic METH treatment increased, whereas chronic haloperidol treatment decreased, *Pdlim5* mRNA levels in the prefrontal cortex. Imipramine increased *Pdlim5* mRNA levels in the hippocampus.

**Conclusion:**

These findings are partially compatible with reported observations in humans, indicating that PDLIM5 is involved in psychiatric disorders, including mood disorders.

## Introduction

The *PDLIM5* gene encodes the enigma homolog (ENH). In total, 4 known isoforms of the human PDLIM5 protein have been identified: ENH1, ENH2, ENH3, and ENH4. The largest isoform, ENH1, contains 1 PDZ (postsynaptic density-95/discs large/zone occludens-1) domain and 3 LIM (Lin-11, Isl-1, and Mec-3) domains at the C-terminal end [1]. ENH1 is expressed in various tissues, such as the heart, brain, spleen, liver and kidney. In comparison, shorter isoforms that lack the LIM motifs are expressed in cardiac (ENH3) and skeletal muscle (ENH2, ENH3, and ENH4) [1], [2], [3]. ENH1 is expressed in various regions of the brain, including the hippocampus, cortex, thalamus, hypothalamus, amygdala, and cerebellum [4].

ENH1 was originally identified as a PKC-interacting protein [1] and which may activate conventional PKCs by directly binding them through its LIM domains [5]. The ENH1 LIM domain is one of the targets of protein kinase C (PKC), which has been shown to bind to the regulatory domain of PKCβ1 and ε· [1], [4]. ENH has been shown to be localized in the presynaptic nerve terminals [4], [6] and in the postsynaptic density [7]. ENH has been reported to interact with CaV2.2, which is a presynaptic calcium channel type that is known to be sensitive to enhancement by PKC [4] and may be an adaptor for the regulation of intracellular calcium levels by constructing a PKCε–ENH–N-type Ca^2+^ channel complex [8]. However, the mechanism by which ENH and Cav2.2 interact remains controversial [6]. In the postsynaptic density, PDLIM5 is reported to interact with spine-associated RapGAP (SPAR, SIPA1L1), and to promote the shrinkage of dendritic spines [7]. RNA interference against PDLIM5, or loss of PDLIM5 interaction with SPAR, causes an increase in spine head diameter. PKC activation promotes the delivery of PDLIM5 into the dendritic spines and increases its spine co-localization with SPAR [7].

Recent studies have shown that SNPs in the *PDLIM5* gene are associated with schizophrenia [9], [10], [11], bipolar disorder [10], [12], [13], and major depression [14], [15]. In contrast, other studies have reported no significant genetic association between the *PDLIM5* gene and schizophrenia [16], [17]. However, the associations have been replicated in independent populations with bipolar disorders [12], [13] and major depression [15].

The precise molecular roles of PDLIM5 in mood disorders remain unclear. An association between the SNP rs2433320 and bipolar disorders confirmed by a meta-analysis [12] was also observed in the genome-wide association study carried out by The Wellcome Trust Case Control Consortium (WTCCC) (*p* = 0.03) [18]. The SNP rs2433320, which is located approximately 2 kb upstream of *PDLIM5*, was reported to be associated with *PDLIM5* mRNA levels in the prefrontal area [9]. Iwamoto and colleagues used DNA chip analysis to examine the prefrontal regions of postmortem brains of patients with mental disorders, and found that *PDLIM5* was upregulated in patients with schizophrenia, bipolar disorder and major depression [19]. The upregulation of *PDLIM5* in the prefrontal cortex and cerebellum has also been show by the Stanley Medical Research Institute Online Genomics Database for schizophrenia (*p* = 0.037) and for bipolar disorder (*p* = 0.38), though it did not show significant upregulation for bipolar disorder (https://www.stanleygenomics.org/stanley/geneDetail.jsp?gene_id=7370). The *PDLIM5* mRNA expression levels in the peripheral leukocytes of medication-free patients with schizophrenia were significantly higher than in those of control subjects [16]. In contrast, decreased peripheral *PDLIM5* mRNA expression in whole blood from manic patients with bipolar disorder type I was reported [20]. The authors also reported no significant difference in *PDLIM5* mRNA expression following treatment with olanzapine treatment while reducing in the severity of manic symptoms [20]. In addition, significantly lower *PDLIM5* mRNA expression levels have been reported in the peripheral leukocytes of drug-naïve patients with depression than in those of control subjects. Furthermore, after 4 weeks of paroxetine treatments, *PDLIM5* mRNA expression significantly increased to almost the same level as the control subjects [17]. Thus, studies on *PDLIM5* expression have shown variable results when using postmortem brains and peripheral leukocytes in patients with mood disorders. It should be noted that there is a difference in the expression level of the *PDLIM5* gene in the peripheral blood cells of patients with major depressive disorder and schizophrenia who were undergoing treatment with antidepressants and antipsychotics [16], [17].

In terms of PKC activity, which is mutually regulated by ENH1, the prefrontal cortex of postmortem subjects with major depression exhibited significantly lower protein expression of PKCβ1 and PKCε compared to controls [21]. Postmortem studies have demonstrated that membrane-associated PKC and the stimulation-induced translocation of the cytosolic enzyme to the membrane increased in the frontal cortex of patients with bipolar disorder [22], [23]. A significant increase in membrane-associated platelet PKC activity has been demonstrated in manic patients, while enhanced PKC activity during mania is suppressed by mood-stabilizer treatments [24], [25], [26]. In comparison, decreased PKC activity and PKCβI and βII levels, but not PKCα or PKCζ, in the membrane and cytosol fractions of platelets from medication-free pediatric patients with bipolar disorder has been reported [27]. In general, many other studies support the involvement of PKC and its substrates in the pathophysiology of bipolar disorders and their use in the treatment of bipolar disorders [28], [29], [30], [31], [32].

Thus, the links between PDLIM5 expression and mood disorders, and between PKC and mood disorders, have been reported in humans, while the link between PDLIM5 and PKC has been reported for cell and in-vitro experiments. However, the experimental analysis of lowered *Pdlim5* expression and the disruption of PKCε translocation on mood disorder in mouse models have not been reported. To obtain insights about the effects of PDLIM5 expression on the whole body of individuals with mood disorders, we examined the effects of constitutionally reduced levels of the ENH1 isoform of PDLIM5 generated by the ENH1 isoform of *Pdlim5* knockout (KO) mice on behaviors associated with mania and depression. Because it has been acknowledged that single-dose methamphetamine (METH) treatment and chronic METH treatment may model mania and the evolution of mania into psychotic mania or schizophrenia-like behavioral changes, respectively [33], we evaluated behaviors in response to METH treatments in *Pdlim5* hetero KO mice. The mood stabilizers lithium and valproate are known to reverse METH-induced hyperactivity and behavioral sensitization [34], [35], [36].

In addition, we evaluated the effects of administrating the PKCε-translocation inhibitor peptide (PKCε-TIP) on the response of mice to METH. The LIM domains of PDLIM5 have been identified as interaction sites for various PKCβI and ε isoforms, while the V1 region of PKC has been identified as critical for PDLIM5-PKC interaction [1]. PKCε-TIP, which is a small water-soluble peptide (8 amino acid residues) derived from the V1 region of PKCε [37], may specifically inhibit the effects of PKCε by blocking the translocation of PKCε from the cytosol to the membrane [4]. PKCε-TIP has been used to specifically inhibit the effects of PKCε in in vivo experiments [38].

As a parameter of depressive behavior, we examined the immobilization of *Pdlim5* hetero KO mice in a forced swimming test, with and without the administration of imipramine.

## Materials and Methods

### Generation of Pdlim5-deficient Mice and the Mice Used in This Study

**Figure 1:**
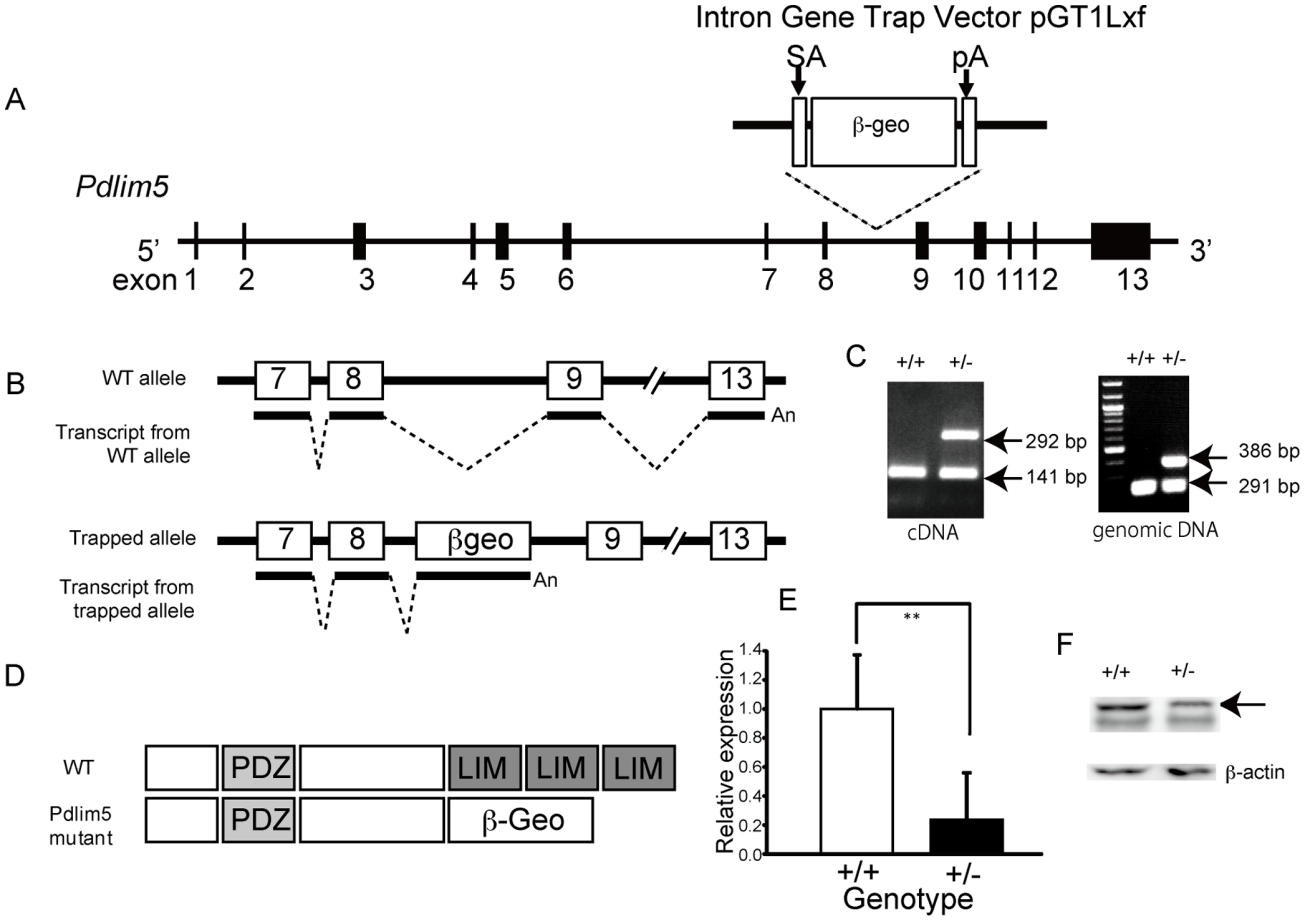
Gene-trap mutagenesis of *Pdlim5*. (A) Insertion site of the gene-trap cassette in intron 8 of the *Pdlim5* gene. The inserted sequence (gray box) includes a splice acceptor (SA), β-geo, which is a fusion of β-galactosidase and neomycin phosphotransferase II, and is followed by a stop codon and a polyadenylation signal (pA). (B) Schematic of the gene trap cassette. C) RT-PCR results and genomic genotyping of *Pdlim5+/+*, *Pdlim5+/−*. Samples were extracted from the brain and tail. (D) Expected domain structures of wild-type and PDLIM5 mutant proteins. (E) Real-time PCR analysis of *Pdlim5* in the prefrontal brains of *Pdlim5*+/+ (n = 6) and *Pdlim5*+/− (n = 8) mice [*F*(1, 12) = 14.0, *p* = 0.003]. Values are shown as mean ± SD. (F) Representative western blots of *Pdlim5* in the prefrontal brains of *Pdlim5*+/+ and *Pdlim5*+/−.

The mouse *Pdlim5* gene-trapped ES cell line (XH199) was obtained from the Mutant Mouse Regional Resource Center (http://www.mmrrc.org/). The *Pdlim5* gene, which contains 13 exons, specifies a PDZ domain and 3 LIM domains. Exon 2 of *Pdlim5* specifies the end of the PDZ domain, while exon 9 specifies the beginning of the first LIM domain. The gene-trap vector, pGT1lxf containing the *Engrailed 2* (En2) splice acceptor sequence, β-*geo* (β-galactosidase/neomycin phosphotransferase fusion gene) and a SV40 poly (A) signal sequence generated a mature *Pdlim5* (exons 1–8)- β-*geo* mRNA, which was determined by 5′ RACE PCR at BayGenomics (http://baygenomics.ucsf.edu/). Thus, incorporation of the gene trap generates a fusion between the PDZ and the β-*geo* gene products (Fig. 1A–D), which contains 1–368 amino acids (aa) of the ENH1 isoform and 1–306 aa of the ENH2 isoform. The ES cell line was injected into C57BL/6J host blastocysts to generate chimeras. The chimeras were then crossed with C57BL/6J mice to determine germ line transmission. Total RNA and DNA were extracted from the tail tissues by using ISOGEN (WAKO, Tokyo, Japan). cDNA was synthesized using ReverTra Ace (Toyobo, Tokyo, Japan) and oligo dT primers. Genotype detection was carried out by PCR of cDNA by using 3 primer methods. To detect the 5′ end of the gene trap insertion, the primers used were as follows: *Pdlim5*_exon8Fc: 5′-CACCAGCGTCAAGTCACCTA-3′, bgeoRc: 5′-ATTCAGGCTGCGCAACTGTTGGG-3′, and *Pdlim5*_exon9Rc: 5′-TGCACAAGTGTGTCTTGGTCAC-3′. Routine genotyping was carried out by PCR of genomic DNA by using the following primers: bgeoR: 5′-ATTCAGGCTGCGCAACTGTT-3′, *Pdlim5*_ex8F: 5′-CACCAGCGTCAAGTCACCTA-3′, *Pdlim5*_exon9R: 5′-TGCACAAGTGTGTCTTGGTCAC-3′, and *Pdlim5*_exon9F: 5′-CCGCCTAGTGTTTGCATCATAA-3′.

The animals were housed under a 12-h light/12-h dark cycle, with constant temperature (25°C) and humidity, and were allowed free access to food and water. All animal procedures were performed according to protocols approved by the Animal Care and Use Committee of the University of Tsukuba, Japan.

Unless otherwise specified, 6- to 8-week-old male mice were used in this study; the mice were derived from 6 to 8 backcrosses (N6∼N8) to C57BL/6J (Charles River Laboratories Japan, Yokohama, Japan) from the chimera (129 X C57BL/6). Each behavioral experiment was performed independently using a new group of animals, except for novelty-seeking, rota-rod, social interaction, and prepulse inhibition tests without drug administration. These tests were performed consecutively every week in this order, using mice of the N6 backcross generation; however, the number of mice tested differed among each of these tests.

### Analysis of *Pdlim5* Transcription in the Brain Tissue of Mice

The prefrontal cortex, midbrain, hippocampus, thalamus, and striatum were removed by dissection. Total RNA was then extracted from the brain tissues with ISOGEN Reagent (Nippon Gene Co, Tokyo, Japan). *Pdlim5* cDNA was synthesized with ReverTra Ace (Toyobo) and the oligo dT primer from RNA. Expression was quantified by real-time quantitative PCR analysis with the TaqMan Gene Expression Assay and ABI PRISM 7900HT Sequence Detection System (Applied Biosystems, Foster City, CA), according to the manufacturer’s instructions. Primers and probes were purchased from Applied Biosystems (Assay ID: Mm00517301_m1). Rodent glyceraldehyde-3-phosphate dehydrogenase (*Gapdh*) was used as an internal control, and the threshold cycle (Ct) was measured in triplicate. Data were collected and analyzed with Sequence Detector Software (SDS) version 2.2.2 (Applied Biosystems). The relative gene expression was calculated as the ratio of *Pdlim5* to the internal control *Gapdh*. The Ct measurement was the average of 3 replicates. Six wild-type and 8 heterozygous, 8-week-old male mice of the N3 backcross generation were used in this *Pdlim5* transcriptional analysis.

### Western Blot Analysis

Protein was extracted from brain tissue with Laemmli buffer. The concentration of total protein was measured by using a Wallac 1420 ARVOsx multilabel counter (Perkin Elmer, Yokohama, Japan). Then, 2 µg of each sample was run on a Pro-Pure™ SPRINT NEXT GEL (Amresco, Solon, OH) and transferred to BioTrace™ PVDF (Nihon Pall Ltd., Tokyo, Japan). Human PDLIM5 Polyclonal Antibody (1∶100dilution) (MBL, Nagoya, Japan) was used as the primary antibody, or polyclonal antibody to beta-Actin (1∶500 dilution) (Imgenex, San Diego, CA) for normalization. Bound primary antibodies were detected with goat anti-rabbit IgG antibody HRP conjugate (MBL) in 1∶10000 dilution and Immobilon™ Western Chemiluminescent HRP Substrate (Millipore, Billerica, MA) by LAS-4000UVmini (Fujifilm, Tokyo, Japan). LAS-4000UVmini directly measures the amount of both proteins on the membrane, and facilitates the calculation of relative *Pdlim5* expression among subjects. Six wild-type and 8 heterozygous, 8-week-old mice of the N10 backcross generation were used in this *Pdlim5* western blot analysis.

### Drugs and Administration Schedule

METH (Dainippon Sumitomo Pharma Co., Ltd, Osaka, Japan) was dissolved in saline solution. The acute METH treatment group received a single dose of METH injection (3 mg/kg, intraperitoneal [i.p.]). The chronic METH treatment group was generated by the injection of 1 mg/kg METH after i.p. injection of 3 mg/kg METH once daily for 14 days and a subsequent 14-day withdrawal period. This chronic treatment developed behavioral sensitization to METH, which was confirmed by 1 mg/kg injection of METH. Haloperidol was dissolved in 0.9% saline solution with tiny drops of lactate, and was administered (1 mg/kg, i.p.) daily for 7 weeks. Imipramine was dissolved in saline solution and administered (20 mg/kg, i.p.) daily for 14 days. The control group for each experiment received saline injections.

### Locomotor Activity Analysis

To evaluate the behavioral effects of METH treatment, spontaneous horizontal locomotor activity was measured using the Lime Light (Actimetrics, Wilmette, IL) CCD camera tracking system. The mice were placed inside soundproof and illuminated plastic cages (40 cm × 20 cm × 20 cm). Each mouse was placed separately in a box for a 60-min habituation period. Spontaneous activity was recorded for 60 min (habituation to the experimental procedure), and then the mice were injected with i.p. METH or saline solution, and locomotor activity was recorded for an additional 120 min immediately after injection. The total distance traveled was recorded. The effects of acute METH treatment on locomotor activity were measured for 13 wild-type and 13 heterozygous mice, and the effects of chronic METH treatment on locomotor activity was measured for 11 wild-type and 23 heterozygous mice. Locomotor activity without drug treatment was measured in 4 wild-type and 6 heterozygous mice.

### Acoustic Startle Response (Prepulse Inhibition, PPI) Test

PPI refers to the reduction in the startle response produced by the antecedent presentation of a low-intensity non-startling stimulus, representing sensorimotor gating [39]. Sensorimotor gating deficits in patients with schizophrenia, as indexed by measures of PPI, have been well characterized and are suggested as meeting the criteria of a useful endophenotype in human genetic studies [40]. With respect to bipolar disorder, current research suggests that PPI deficiency is state dependent rather than a constant trait. Patients with bipolar disorder with acute mania symptoms and psychosis exhibit PPI deficits [41]. PPI is measured using an SR-Lab system (San Diego Instruments Inc., San Diego, USA). Wild-type and hetero KO male mice (age, 6–8 weeks) were pre-treated intraperitoneally with saline or METH (1 mg/kg) 5 min before testing. After treatment, they were placed in a Plexiglas cylinder test chamber. Experimental sessions consisted of a 5-min acclimatization period with 70-dB broadband background noise, which was continuous throughout the session, followed by three 120-dB acoustic pulses and PPI sessions. Sessions consisted of 10 blocks of 5 different trial types: startle pulse alone with a 40-ms duration at 120 dB (P120); and 4 prepulse+pulse trials with a 20 ms duration prepulse at either 78 dB (PP78), 82 dB (PP82), or 86 dB (PP86) followed by a 40-ms duration startle stimulus at 120 dB after a 100-ms delay. These were carried out in a pseudorandom order, with an average intertrial interval of 15 s. The startle response was recorded as maximum startle amplitude. PPI was calculated as a percentage score for each prepulse intensity, using the following equation: %PPI = 100× [(mean startle amplitude on pulse-alone trials – mean startle amplitude on prepulse trials)/mean startle amplitude on pulse-alone trials]. The startle magnitude was calculated as the average of all pulse-alone (p120) trials, excluding the first 3 p120 trials in each session. The effects of acute METH treatment on PPI were measured in 11 wild-type and 13 heterozygous mice, and the effects of chronic METH treatment on PPI was measured for 12 wild-type and 24 heterozygous mice. PPI without drug administration was measured for 10 wild-type and 10 heterozygous mice.

### Forced Swimming Test

The forced swimming test was performed according to the method of Porsolt et al. (1977), with minor changes. The day before the test, mice were anesthetized with isoflurane, and wire rings (0.28 mm in diameter, 1.5 cm in length, and 10 mg in weight) were attached to both hind paws. A small magnet (1 mm in diameter, 3 mm in length) was attached to each of the wire rings. After a 12- to 18-h habituation, mice were subjected to forced swimming tests. Narrow plastic cylinders (18 cm in height, 11 cm in diameter) were filled with water (maintained at 25±1°C) to a depth of 10 cm. The cylinders were located in a MicroAct (Neuroscience Inc., Tokyo, Japan; see Inagaki et al. 2002) behavioral measuring system, which allowed the detection of motion by registering electric currents induced in a magnetic coil surrounding the cylinder by using magnets attached to the limbs of the mice. Mice were placed in the cylinders and motion was recorded for 6 min. The mice were judged immobile whenever they floated passively in the water; the judgment was based on threshold values. Electrical signals meeting the following criteria were classified as swimming: signals greater than 100 mV that continued for more than 0.1 s without gaps of more than 1.0 s. Immobility time was measured during the last 4 min of the 6-min session. Imipramine (20 mg/kg) or saline was injected for 14 days, and the test was performed 40 min after the last injection. The forced swimming test was performed on 24 wild-type and 9 heterozygous, 8-week-old male mice of the N8 backcross generation after saline injection, and on 12 wild-type and 12 heterozygous mice after imipramine injection.

### Rota-rod, Novelty-seeking, and Social Interaction Tests

Rota-rod, novelty-seeking, and social interaction tests were carried out according to the methods described in the published literature [42], [43]. The rota-rod tests were performed in 10 wild-type and 20 heterozygous mice, the novelty-seeking tests were performed on 24 wild-type and 41 heterozygous mice, and the social interaction tests were performed on 8 wild-type and 21 heterozygous mice.

### Surgery and Intracerebroventricular Infusion Procedure

Mice were anesthetized with sodium pentobarbital (40 mg/kg, i.p.), and mounted in a stereotaxic frame. A stainless steel guide cannula (23 gauge) was placed into the lateral ventricle (AP, 0.46 mm; ML, 1.01 mm to the bregma) for subsequent intracerebroventricular (icv) infusion. The guide cannula extended 4.0 mm below the surface of the skull. One Anchor Screw (EICOM, Kyoto, Japan) was placed into the skull and dental cement was poured over the exposed skull to hold the screws and cannula in place and close the incision site. A dummy cannula was placed into the guide. Mice were allowed to recover for 3 days prior to testing. Cannula placement was assessed in random animals via the icv infusion of xylene cyanol and the verification of dye in the ventricular system.

During infusions, the dummy cannulas were removed. An infusion cannula (30 gauge), which was attached to PE 20 tubing, was inserted into the guide cannula. A 10.0-µl Hamilton syringe was used to manually deliver Ringer’s solution or PKCε-TIP (Merck KGaA, Darmstadt, Germany) over a 1-minute period. The infusion cannula was kept in place for an additional 30 s following infusion. Mice were then replaced into their original cages. The change in locomotor activity induced by METH injection was measured in 6 mice infused with Ringer’s solution infusion and 10 mice infused with PKCε-TIP.

### Statistical Analysis

Data were analyzed using JMP software version 7.0.1 (SAS Institute, Cary, NC, USA). The data of the expression analysis were analyzed by one-way analysis of variance (ANOVA) followed by post hoc analysis using a Student’s *t* test to evaluate the effect of METH or imipramine administration in each brain area. Effects of genotype, METH treatment, and imipramine treatment on behavior were analyzed by ANOVA, followed by post hoc analysis using a Student’s *t* test. The results were expressed as the mean ± SD. A *p* value <0.05 was considered significant.

## Results

### *Pdlim5* Deficient Mice

To generate *Pdlim5* KO mice, *Pdlim5*+/− mice were crossed; however, *Pdlim5* KO mice were not identified among 134 genotyped pups, indicating that null *Pdlim5* results in embryonic lethality. No apparent phenotypic differences were observed between the wild-type and *Pdlim5* hetero KO mice. No significant difference in body weight, brain size, or brain shape was observed between *Pdlim5*+/− and *Pdlim5*+/+ mice. *Pdlim5* expression levels in the prefrontal cortex of hetero KO mice (*Pdlim5*+/−) were less than 50% of those in the wild littermate mice (*Pdlim5*+/+) (Fig. 1E). The expression of PDLIM5 protein decreased in *Pdlim5*+/− compared to the wild littermate mice (*Pdlim5*+/+) (Fig. 1F).

### Behavioral Characteristics of *Pdlim5* Deficient Mice

No significant difference in rota-rod (Fig. S1A), novelty-seeking (Fig. S1B), social interaction (Fig. S1C), locomotion (Fig. S1D), or PPI tests (Fig. S1E) was observed between *Pdlim5*+/+ and *Pdlim5*+/− mice.

### *Pdlim5*+/− Mice in Mania Models

**Figure 2:**
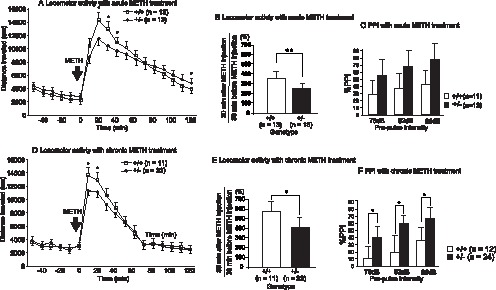
Effect of METH administration on locomotor activity and prepulse inhibition. Relative locomotor activity and prepulse inhibition after acute (A, B, C) and chronic (D, E, F) administration of METH in *Pdlim5*+/+ and *Pdlim5*+/− mice. Values are shown as mean ± SD. * *p*<0.05 and ** *p*<0.01 by ANOVA.

Single-dose METH treatment produced an enhancing effect on spontaneous locomotor activity in mice. The magnitude of enhanced locomotor activity by an single-dose injection of METH was significantly lower in *Pdlim5*+/− mice than in *Pdlim5*+/+ mice when comparing locomotor activities 30 minutes before and after METH injection [*F*(1, 24) = 9.4, *p* = 0.005] (Fig. 2A, 2B). An injection of METH (3 mg/kg) impaired PPI in *Pdlim5*+/+ mice, but did not appear to impair PPI in *Pdlim5*+/− mice, leading to significantly less impairment of PPI in the acoustic startle response of *Pdlim5*+/+ mice compared to *Pdlim5*+/− mice; ANOVA revealed a significant main effect of genotype [*F*(1, 70) = 5.1, *p* = 0.03] (Fig. 2C). No significant difference was observed between the Pdlim5+/− and Pdlim5+/+ mice for the startle response to 120-dB acoustic stimuli when treated with saline or acute or chronic METH treatment (data not shown).

The effect of the METH injection (1 mg/kg), after 14-day treatment with METH (3 mg/kg) and 14 days of subsequent withdrawal, was also significantly lower in *Pdlim5*+/− mice than in *Pdlim5*+/+ mice when comparing locomotor activities 30 min before and after METH injection [*F*(1, 32) = 6.2, *p* = 0.02] (Fig. 2D, 2E). An injection of METH (1 mg/kg) also produced significantly less impairment of the PPI in the acoustic startle response of *Pdlim5*+/− mice compared to *Pdlim5*+/+ mice; ANOVA revealed a significant main effect of the genotype [*F*(1, 106) = 9.3, *p* = 0.003] (Fig. 2F).

### Effects of PKCε-TIP on Locomotor Activity and PPI Response to METH

**Figure 3:**
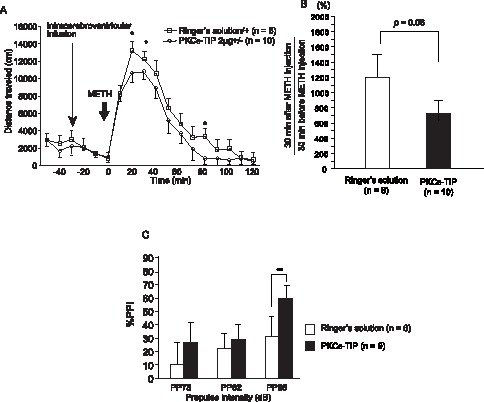
Effects of PKCε-TIP on locomotor activity and prepulse inhibition response to METH. Relative locomotor activity (A, B) and prepulse inhibition (C) by the treatment of PKCε-TIP after chronic METH administration. Values are shown as mean ± SD. P values are based on ANOVA. * p<0.05 and ** p<0.01 by ANOVA.

After being treated with 3 mg/kg METH (i.p.) for 14 consecutive days followed by 14 days of withdrawal, 2 µg of PKCε-TIP or Ringer’s solution was injected via icv fusion for 7 consecutive days. After this treatment, a 1 mg/kg METH challenge was provided intraperitoneally on the 8th day. PKCε-TIP treatment produced a weakened locomotor response to METH injection compared to Ringer’s solution treatment [*F*(1,14) = 4.1, *p* = 0.06] (Fig. 3A, 3B). PKCε-TIP treatment produced significantly less impairment of the PPI in the acoustic startle response compared to treatment with Ringer’s solution [*F*(1,43) = 4.54, *p*<0.05] (Fig. 3C).

### *Pdlim5*+/− Mice in the Forced Swimming Test and the Effect of Imipramine Treatment

**Figure 4:**
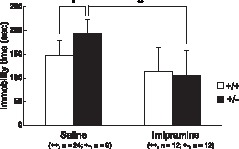
Effect of imipramine treatment on forced swimming. Immobility time of *Pdlim5* hetero KO and wild-type mice in the forced swimming test with saline or chronic imipramine administration. Values are shown as mean ± SD. * p<0.05 and ** p<0.01 by ANOVA.

We conducted a forced swimming test, which is a common behavioral test for assessing depression in rodents and for testing the efficiency of antidepressant drugs, in *Pdlim5* hetero KO mice. A significant increase in immobility time was observed in *Pdlim5* hetero KO mice compared to the wild-type mice when they were treated with saline [*F*(1,31) = 7.3, *p* = 0.01]. Imipramine significantly attenuated immobility time in *Pdlim5* hetero KO mice [*F*(1, 16) = 12.0, *p* = 0.003]. A decrease in immobility time was also observed in wild-type mice, but was not significant [*F*(1,33) = 1.5, *p*>0.05] (Fig. 4).

### Changes in *Pdlim5* Expression Levels in Mouse Brains Following METH, Haloperidol, and Imipramine Treatment

**Figure 5:**
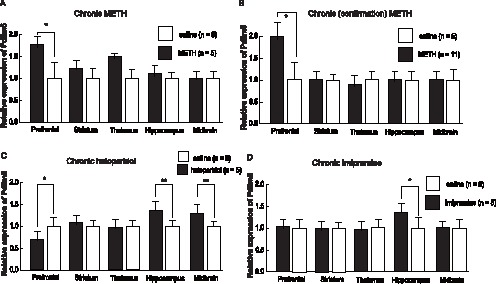
Relative Pdlim5 expression levels after METH, haloperidol and imipramine administrations in mice brains. Seven-week-old C57BL/6J male mice were treated with an intraperitoneal injection (i.p.) of METH (3.0 mg/kg, once daily for 14 days) (A, B), haloperidol (1 mg/kg, once daily for 49 days) (C), imipramine (20 mg/kg, once daily for 14 days) (D), or vehicle-saline. The upregulated *Pdlim5* expression in the prefrontal cortex of chronic METH-administered mice (A) was confirmed by a separate experiment using a different mice cohort (B). Values are shown as mean ± SD. * *p*<0.05 and ** *p*<0.01 by Student’s t test.

We investigated whether chronic METH administration induces changes in *Pdlim5* expression in mouse brains. *Pdlim5* expression levels in the brains were not significantly different in 7-week-old male mice (C57BL/6J) sacrificed 24 h after 1 dose of i.p. METH injection (3 mg/kg) or in saline-injected mice [*F*(5, 44) = 1.9, *p* = 0.11]. *Pdlim5* expression levels in the brains were significantly different in mice 24 h after administering the last chronic injection (3 mg/kg for 14 consecutive days) and mice injected intraperitoneally with saline [*F*(5,44) = 4.8, *p* = 0.001]. Post hoc analysis revealed higher *Pdlim5* expression levels in the prefrontal area of chronic METH-administered mice than in saline-administered mice (*p* = 0.03; Fig. 5A). The upregulated *Pdlim5* expression in the prefrontal cortex of chronic METH-administered mice was confirmed by a separate experiment with different mice (*p* = 0.01) (Fig. 5B).

Chronic administration of haloperidol did not produce a significant difference in *Pdlim5* expression levels in the overall brain of mice sacrificed 24 h after administering the last chronic injection (1 mg/kg for 49 consecutive days) or in mice injected intraperitoneally with saline [*F*(5,93) = 1.3, *p* = 0.27]. However, post hoc analysis revealed lower *Pdlim5* expression levels in the prefrontal area of mice, and higher *Pdlim5* expression levels in the hippocampus and midbrain after chronic haloperidol administration compared to mice administered with saline (*p* = 0.05, 0.009, and 0.06, respectively; Fig. 5C).

*Pdlim5* expression levels in the brains were significantly different for mice after chronic imipramine administration compared to mice after 14 days of i.p. saline injection [*F*(5,45) = 6.3, *p* = 0.0002]. Post hoc analysis revealed higher *Pdlim5* expression levels in the hippocampal area of chronic imipramine-administered mice than in saline-administered mice (*p* = 0.03; Fig. 5D). *Pdlim5* expression levels in the brains were not significantly different for mice administered 1 dose of imipramine compared to saline-administered mice [*F*(1, 44) = 1.9, *p* = 0.11].

## Discussion

To our knowledge, this is the first report about *Pdlim5* KO mice. The removal of exons 9–13 eliminates binding determinates for the LIM domain, consequently disrupting the interaction of PKC. The targeted *Pdlim5* allele generated mice that expressed a fusion protein lacking the LIM domain, thereby allowing the physiological role of the PDLIM5 LIM domain to be assessed. LIM domains are members of the zinc-binding domain family that is found in many key regulators of developmental pathways. The present study identified that embryonic lethality occurs when the PDLIM5 LIM domain is lost in mice. PDLIM5 is expressed in various tissues, including the heart, skeletal muscle, brain, lung, spleen, thymus, prostate, testis, ovary, small intestine, colon, leukocyte, liver, and kidney [3]. PDLIM5 preferentially interacts with PKCβ through the LIM domains in cardiomyocytes [1], and has an important role in heart development by scaffolding PKCβ to the Z-disk region [44]. It also binds to PKD1 and interacts with the cardiac L-type voltage-gated calcium channel subunit alpha1c in cardiomyocytes [45]. The silencing of *Pdlim5* inhibits the alpha-adrenergic-induced increase of L-type calcium currents [45]. Although we did not examine *Pdlim5*−/− embryos, the lethal embryonic phenotypes of *Pdlim5*−/− mice may have arisen because of embryonic heart/circulation failure. In zebrafish, ENH knockdown appears to have a lethal effect on embryos shortly after the end of gastrulation, with some embryos showing elongation defects and disorganized somites [46]. This observation indicates that PDLIM5 has a crucial role in embryonic development.

This study aimed to evaluate the effects of decreased PDLIM5 levels on certain behaviors, specifically those related to mood disorders, because significant but not robust evidence for mood disorders in humans has been reported. We used *Pdlim5* hetero KO mice, because an increase or reduction of *PDLIM5* expression in human brains, peripheral lymphoblastoid cells, and lymphocytes has been reported to be linked with schizophrenia and mood disorders [16], [17], [19], [47]. Because differences in the size and weight of the brain and body, or other anatomical abnormalities, were not found in the present study in *Pdlim5* hetero KO C57BL/6J mice, haploinsufficiency of *Pdlim5* did not appear to cause the developmental abnormalities. In addition, the behavior tests (rota-rod, novelty-seeking, and social interaction tests) did not show any significant difference in locomotion between *Pdlim5*+/+ and *Pdlim5*+/− mice. Therefore, decreased *Pdlim5* expression is unlikely to have a major effect on mouse behavior. However, when mice were treated with METH in the present study, significant differences in locomotion and PPI were observed. For instance, acute and chronic METH-induced hyperlocomotion and PPI were less severe in *Pdlim5*+/− than in *Pdlim5*+/+ mice. These findings suggest that lower levels of PDLIM5 prevent the development of a manic state or schizophrenia, which is consistent with the speculation from the observations in human postmortem brains, because higher levels of PDLIM5 have been reported in the prefrontal cortex of patients with schizophrenia and bipolar disorders [10], [19]. The mechanisms for these findings are subject to speculation. However, the transient inhibition of PKCε by pretreatment with PKCε-TIP produced similar effects on locomotion to those observed in *Pdlim5* hetero KO mice in response to METH. These observations support the speculation that the involvement of PDLIM5 in the behavioral response to METH is partly via calcium or PKCε signaling.

A significant increase in the immobility time of forced swimming test was observed in *Pdlim5* hetero KO mice compared to the wild-type mice. This observation could also be interpreted as being consistent with observations in human peripheral leukocytes. Iga and colleagues also reported that decreased *PDLIM5* levels returned to normal after 8 weeks of medication in the peripheral lymphocytes of patients with depression [17].

The same group of authors reported an increased expression of *PDLIM5* in peripheral leukocytes in medication-free patients with schizophrenia but not in patients administered antipsychotic medication [16]. These reports raise the possibility that antipsychotics reduce *PDLIM5* expression levels, while antidepressants increase *PDLIM5* expression levels. Such a possibility is partially supported by the findings obtained through the chronic administration of haloperidol and imipramine; haloperidol decreased *Pdlim5* expression levels in the prefrontal area, while chronic administration of imipramine increased *Pdlim5* expression levels in the hippocampus area. However, chronic administration of haloperidol also increased *Pdlim5* expression levels in the hippocampus and midbrain, indicating the presence of a complex relationship between drugs and *Pdlim5* expression. An alternative possibility is that these drugs do not directly influence *PDLIM5* expression, but that *PDLIM5* levels are related to the psychiatric state.

PDLIM5 may also be implicated in neural development. For instance, in the present study, *Pdlim5* expression levels increased in the hippocampus of mice receiving chronic haloperidol or imipramine treatment compared to the control. Adult hippocampal neurogenesis has been implicated in antidepressant action [48]. Adult treatment with haloperidol is reported to increase dentate granule cell proliferation in the gerbil hippocampus [49], and to promote the survival of stem cells in the rat hippocampus [50]. Lasorella and colleagues reported that PDLIM5 is upregulated during neural development, and that it contributes to the differentiation of the nervous system through cytoplasmic sequestration of Id2, which binds to the PDLIM5 LIM domains [51]. The findings of the present study may support the possibility that some antipsychotics influence *Pdlim5* expression levels, and that changes in *Pdlim5* expression levels may contribute to the worsening or alleviation of some symptoms. Because we did not examine the brain of *Pdlim5*−/− embryos in this study, the effect of the absence of PDLIM5 LIM domains on brain development should be focused on in future studies.

This study was subject to several limitations, including a lack of analyses of *Pdlim5*−/− mice, a lack of specificity of PKCε-TIP to PDLIM5, a lack of measuring hypothalamic-pituitary-adrenocortical axis activity parallel to the behavioral phenotypes, and particularly limitations of a pharmacological model of bipolar disorder mania by METH administration. Despite these limitations, the present study indicates that the increased expression of PDLIM5 may cause behavioral changes to patients with mania or schizophrenia-like symptoms, whereas a decrease in its expression may cause depression. In conclusion, our findings support that PDLIM5 is involved in psychiatric disorders, including mood disorders.

## Supporting Information

Figure S1 **Behavioral characteristics in** ***Pdlim5*** **hetero knockout mice.** The rota-rod, novelty-seeking, social interaction, locomotion, and prepulse inhibition tests were conducted in *Pdlim5*+/+ and *Pdlim5*+/− mice. These behaviors showed no significant differences between the 2 genotype groups. Values are shown as mean ± SD.
(EPS)
